# Use of Telemedicine and Virtual Care for Remote Treatment in Response to COVID-19 Pandemic

**DOI:** 10.1007/s10916-020-01596-5

**Published:** 2020-06-15

**Authors:** Bokolo Anthony Jnr.

**Affiliations:** Department of Computer Science, Norwegian University of Science and Technology NTNU, NO-7491 Trondheim, Norway

**Keywords:** Medical systems, Telemedicine, Virtual care, Coronavirus disease 2019, Remote treatment, Pandemic

## Abstract

The current coronavirus disease 2019 (COVID-19) pandemic has caused significant strain on medical centers resources. Thus, concerns about the reducing and management of COVID-19 are on the rise, as there is need to provide diagnosis, treatment, monitoring, and follow-ups during the pandemic. Therefore, the COVID-19 pandemic has radically and quickly altered how medical practitioners provide care to patients. Medical centers are now responding to COVID-19 through rapid adoption of digital tools and technologies such as telemedicine and virtual care which refer to the delivery of healthcare services digital or at a distance using Information and Communications Technology (ICT) for treatment of patients. Telemedicine is expected to deliver timely care while minimizing exposure to protect medical practitioners and patients. Accordingly, a rapid literature review was conducted, and 35 research studies published from 2019 to May 2020 were employed to provide theoretical and practical evidence on the significance of using telemedicine and virtual care for remote treatment of patients during the COVID-19 pandemic. This article provides practical guide based on how to use telemedicine and virtual care during the COVID-19 pandemic. This study provides implication on the potentials of consolidating virtual care solutions in the near future towards contributing to integrate digital technologies into healthcare.

## Introduction

Coronavirus disease 2019 (COVID-19) has drastically affected healthcare across the globe. [1]. Evidently, there is a concern about the overloading of the health care capacity [2]. Providing primary healthcare during this pandemic appeared to be challenging as healthcare services are being disrupted due to inadequate protective gears, lockdown, risk of infection spread to patients and medical practitioners [3]. In order to better mitigate and manage the spread of coronavirus hospitals can improve the efficiency of their medical system by replacing a proportion of physical treatments with digital technologies [4]. Accordingly, physicians are providing medical-care remotely using telemedicine and virtual services [5]. These virtual care services provide a variety of non-dispensing functions, enabling physician to provide quality medical-care services during the COVID-19 pandemic. Such services may include review of patient medication histories, health education, health therapy management, and drug use review remotely [5].

Telemedicine and virtual care can be integrated into the healthcare system as an approach to maximize the efficiency of healthcare delivery [1, 6]. It promotes social distancing measure and help medical centers in managing prolonged waiting times and risk of disease progression [7]. By minimizing in-person visits and reducing face-to-face contact among physicians and patients, the use of virtual care solutions can help lessen the transmission of the virus and protect medical practitioners from infection [8]. Telemedicine and virtual care can play an important role, especially with successful experiences in the management of previous acute respiratory infections such as Severe acute respiratory syndrome (SARS) and Middle East Respiratory Syndrome (MERS) [9]. Presently, COVID-19 has resulted in many medical centers cancelling and postponing in-person outpatient medical visits [10].

Out of necessity, the use telemedicine and virtual care has been suggested as a method to maintain a continuum of healthcare for patients [9, 11]. As a result, the prevalence of telemedicine and virtual care has rapidly increased during this pandemic. However, only few physicians and patients are adequately educated on how best practice on how to utilize these digital services [12]. Thus, there is need to provide guidelines and recommendation to educate both physicians and patients on how they can best use telemedicine and virtual care [2]. Therefore, the aim of this study is to provide recommendation and guidance to medical practitioners on the use of telemedicine to address COVID-19 response and similar challenges during possible future disasters. The reminder of the article is structured as section 2 is research methodology, section 3 is findings. Section 4 is discussion and section 5 is conclusion.

## Research Methodology

**Fig. 1:**
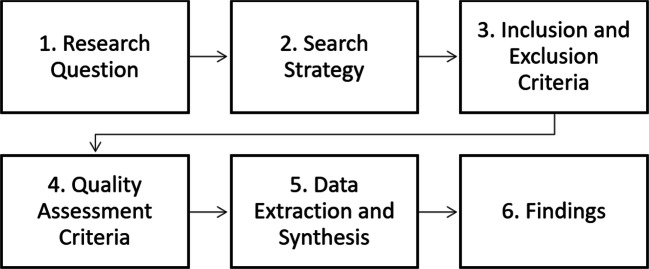
Resign method

This study adopts a rapid literature review methodology to provide evidence similar to prior studies [3, 4, 7, 13]. Figure 1 shows the research method employed to conduct the review.

Figure 1 depicts the research review method employed which comprises of six phases: research questions, search strategy, selection criteria for inclusion and exclusion, quality assessment criteria, data synthesis and extraction, and findings.

### Research Questions

The formulated research questions to be explored in this study are as follows:

- **RQ1**: What are the current legislations to promote deployment of telemedicine and virtual applications during COVID-19 era?
- **RQ2**: What are the guidelines for use of telemedicine and virtual applications?
- **RQ3**: What are the challenges and recommendations towards use of telemedicine and virtual applications?

### Search Strategy

In this study the search strategy was employed to identify studies relevant to provide answers to the research questions. The search strategy was conducted based on the search term and data resources to be researched. Thus, search strings were formulated to execute online search in electronic databases by using keywords strings with Boolean operators (OR/AND) utilized in the search terms. The list of search keyword strings used comprises of telehealth, telemedicine, COVID-19, coronavirus disease 2019, pandemic, digital health, remote care, hospital-at-home, digitalized health care, digitalized medical care, virtual health care, virtual medical care.

In this paper, the relevant studies are retrieved from well-recognized scientific databases and digital libraries selected because they are considered as appropriate libraries for social science, medical science, and information systems. The scientific databases and digital libraries include PubMed, Google scholar, Scopus, Web of science (Clarivate Analytics), ScienceDirect, ProQuest, Emerald, Taylor & Francis, Inderscience, Springer, Sage, ACM, Wiley, and IEEE Xplore. Furthermore, they provide advance search engine with choices for conducting filtered search by keywords via publication category, year and discipline. The search comprises of various types of research publications, such as conference proceedings and journal papers. The searches of relevant literatures were conducted between 8th May 2020 and 15th May 2020.

**Fig. 2:**
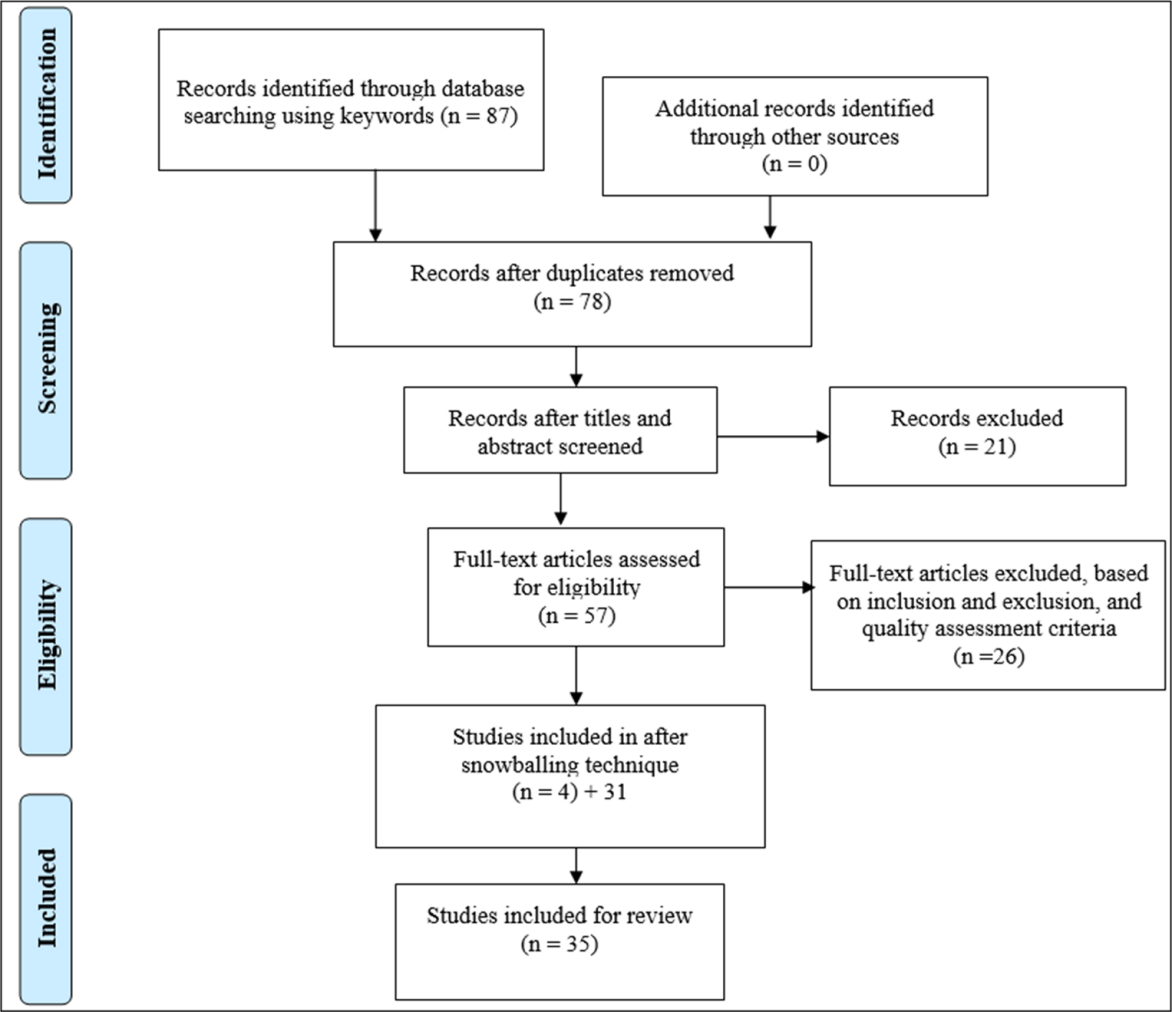
PRISMA flowchart for literature search process

Figure 2 shows the study search phases conducted based on the Preferred Reporting Items for Systematic Reviews and Meta-Analysis (PRISMA) as employed by prior review study on COVID-19 [4, 7]. The search results retrieved 87 articles using the above mentioned keywords. Nine papers were found as duplicates and were removed. Hence, the total number of remaining papers becomes 78. The remaining papers were assessed against the inclusion and exclusion (see Table 1), and quality assessment criteria (see Sect. 2.3). Therefore, 31 articles were found to meet the criteria. After which 4 papers were added based on snowballing technique from cross referencing and a total of 35 papers were included as seen in the reference section.

**Table 1:** Inclusion and exclusion criteria

Inclusion criteria	Exclusion criteria
Studies published in English language that focus of telehealth and COVID-19	Studies that are not written in English language
Journal articles and conference proceedings	Not journal articles or conference proceedings
Published from 2019 till date	Published before 2019
Studies that provide possible answers to research questions based on title and abstract content	Remove duplicate/similar studies by retaining the most current and comprehensive version
Literature review, quantitative, qualitative, trial, and experimental studies that provides evidence	Studies that do not provide any practical, theoretical, trial or statistical evidence

### Inclusion, Exclusion and Quality Assessment Criteria

The list of inclusion and exclusion criteria are presented in Table 1.

One of the important criteria that is required to be checked along with the inclusion and exclusion criteria is the quality assessment. Thus, a quality assessment checklist which evaluates if the selected papers were indexed in ISI Web of Science or Scopus database was employed as a means for evaluating the quality of the studies selected.

### Data Extraction and Synthesis

This stage of the review aims to synthesize and categorize the selected papers based on their scope as related to telemedicine and virtual care platforms during COVID-19 pandemic. Thus, the selected studies were reviewed in detail and relevant data were extracted, analyzed, and synthesized to provide answers to the research questions (see section 2.1). Risk of bias was however not carried out due to the limited available literature in telemedicine for COVID-19 or pandemic at writing of this paper [4].

## Findings

### Legislations that Support Deployment of Telemedicine during COVID-19 Era

Telemedicine is being adopted for triage and treatment of outpatient, enabling protection for patients and medical practitioners to reduce exposure risk from unnecessary exposure [14]. Presently, in order to provide optimal medical-care state the US Department of Health and Human Services (DHHS) and pharmacy boards are temporarily changing requirements and invoking enforcement discretion for telemedicine. Specifically, physicians can use videoconferencing application such as Skype for Business, Microsoft Teams, Updox, or VSee as directed by Health Insurance Portability and Accountability Act (HIPAA). Also, other non-HIPAA compliance tools such as FaceTime, Zoom, Cisco Webex, and Skype are now approved to be temporarily used in telemedicine [14, 15]. Although, Facebook Live or TikTok cannot be used for telemedicine [15–18]. Presently, the Office for Civil Rights at the Department of Health and Human Services in United States (US) is enforcing discretion not to impose penalties for noncompliance with the HIPAA rules [15, 19], to promote the use of audio or video communication platforms during the COVID-19 public health emergency [18].

Moreover, the state pharmacy boards have temporarily permitted pharmacists to remotely work and carryout dispensing activities outside a licensed pharmacy [5]. Furthermore, on March 6th, 2020, The Centers for Medicare & Medicaid Services (CMS) mentioned that it would temporarily pay physicians to provide telemedicine services for beneficiaries across US [9, 14, 17–20]. CMS now allow medical-care providers to utilize devices such as smartphones and electronic devices to treat patients [15, 16, 21–23]. Additionally, on March 17th, 2020 the U.S. Department of Health and Human Services Office for Civil Rights mentioned that during the COVID-19 health emergency, medical practitioners may telemedicine solutions [20], such as MyChart to support patient care [14, 15, 24]. Likewise, the White House Coronavirus Task Force have advised medical-centers to expand their adoption of telemedicine for patient assessment [25].

Furthermore, in the US, virtual medical-care companies such as AmWell and Teladoc have provided communication between patients and physicians through secure video chats. The US government also approved the Coronavirus Preparedness and Response Supplemental Appropriations Act to aid the deployment and use of telemedicine solutions. The law supports use of technologies with audio and video capabilities [26]. The US drug enforcement administration is also allowing medical practitioners to prescribe of medication after patient diagnosis and assessment conducted via telemedicine. Thus, provisionally suspending the Ryan Haight Online Pharmacy Consumer Protection Act of 2008 requirement for an in-person assessment before issuing prescription. Besides, state licensing boards are temporarily removing barriers to inter-jurisdictional telemedicine practice for medical practitioners [25]. Similarly, the American Medical Association has expressed a number of recommendations for ethical practice of telemedicine with which medical practitioners should be familiar before they use telemedicine for treatment [14].

Other countries such as China has been able to control and manage the COVID-19 by using telemedicine. China has managed to minimize the number of new cases since March 2020 by providing health services using virtual care for clinical examinations. For example, the West China Hospital of Sichuan University in collaboration with ZTE China was able to provide remote medical treatment utilizing 5G technology [26]. Likewise, responding to this crisis, the National Telemedicine Center of China (NTCC) located in Zhengzhou, Henan Province established the emergency telemedicine consultation system, which is a telemedicine-based outbreak alert and response system [8]. In conjunction with Huawei Technologies and China Mobile on January 29th, 2020 the NTCC employed 18 workgroups to isolation wards. They helped setup telemedicine equipment and networks. NTCC provides physicians and patients with immediate consultations and diagnosis, regarding COVID-19 [8].

It also provided remote patient monitoring, virtual care, education, and trainings based on interactive live video streaming. Also, prevention and treatment recommendation and guide on drug management and use was provided [8]. A mobile telemedicine device was used to effectively collect, transform, and assess patient health data such as oxygen level, respiratory rate, and blood pressure which reports the data to the attending physician. This helps to prevent direct physical contact, thus decreasing the risk of exposure and prevents potential transmission of infection to nurses and physicians [8]. The Australian government provided funding for Medicare telemedicine services (Medicare support at home) against COVID-19, to encourage physicians to help provide health services. Thus, virtual care visits for all Australians have been expanded to safeguard medical practitioners and patients against the disease [26]. By observing US, China, and Australia’s success in using telemedicine and virtual care to manage COVID-19, other countries can also use telemedicine to manage this pandemic [26].

### Guidelines for Use of Telemedicine and Virtual Applications

**Fig. 3:**
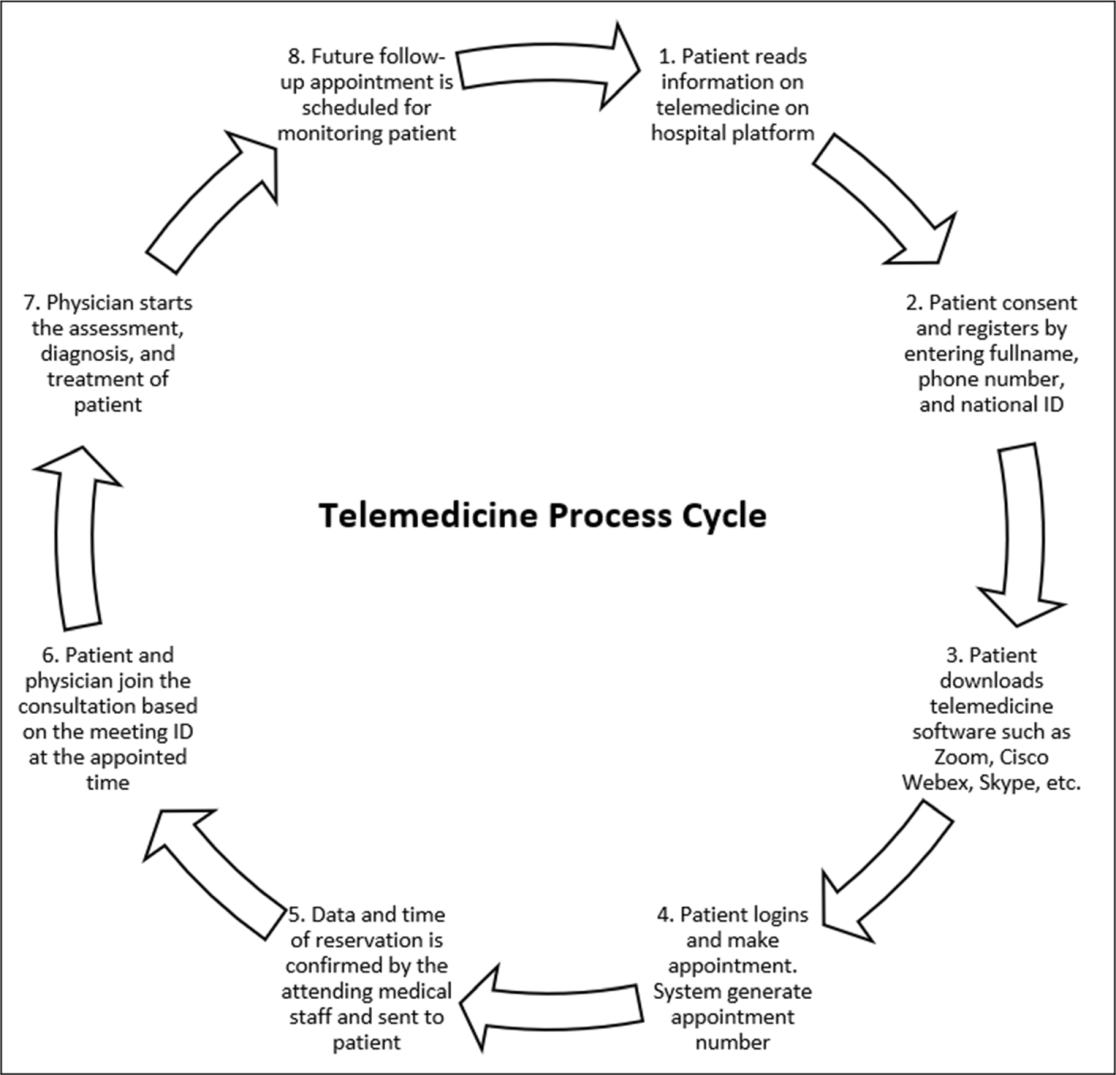
Telemedicine process lifecycle

Telemedicine which is the use of Information and communications technology (ICT) to support and promote long-distance medical-care. Telemedicine entails remote healthcare services and also includes continuing health education, physician training, and administrative meetings [22]. It may also involve the use of existing platforms and systems (such as patient portals) to encourage provide treatment to patients [9]. While telehealth use in hospitals holds great promise, its rapid adoption has created new issues that may impact existing health infrastructure [27]. Hence, there is need to provide guideline to medical practitioners on the use of telemedicine and virtual applications. A few guidelines are among other includes collecting informed consent as it still applies to telemedicine [15, 20, 27], and it is important to point out associated risks that can surface during use of telemedicine to patients during each session when delivering medical-care using remotely. Figure 3 shows a structure of a typical telemedicine process cycle.

Figure 3 depicts the telemedicine process lifecycle. Starting with the patients reading information regarding telemedicine after which the patient consent and registers. Although, conformed consent laws for telemedicine differs widely among countries with some requiring written informed consent and adhering to General Data Protection Regulation (GDPR), while others permit verbal consent. Thus, attending physician should make sure to check their country’s legislation regarding patients consent, confidentiality and privacy agreement [20]. Also, physician must notify patients if any third-party application is being used during virtual consultation and their potential to introduce privacy or cyber security risks. During the online consultation the physician should not let the virtual application get in the way by trying to create the utmost natural environment possible [20]. During each online video session, the physician must dress professionally [2, 17, 20]. Make eye contact with the patient by looking directly to the camera [20]. Although, existing patients have found telemedicine to be an easy extension of the patient/physician relationship. It may sometime take extra effort to make new patients feel comfortable with the virtual treatment process. Thus, physician should try to be friendly and warm and do not make patients feel rushed [20]. Additionally, it is required to obtain background shields as this helps to block background light from patient view.

During the virtual examination, the lighting is very important as the physician being able to see the patient is important. Thus, the patient should ensure that there is proper lighting in the examination positions (e.g., close curtains, add a lamp or light source). Avoid the camera facing a window as it can cause extreme backlighting positioning [27]. Also, physician should confirm that the patient has a working microphones/camera platform [15]. Likewise, it is required for the physician to ensure that telemedicine is within patient medical-care health coverage to verify payment information. If required patients should be trained on how to share their screen and utilize other capabilities of the virtual platform [15]. Professionalism should be upheld throughout the virtual consultation. Physicians should use a high resolution camera from a laptop or an external webcam [2]. Furthermore, physicians should confirm their face is visibly seen in their video and that there is enough lighting.

It may be worthwhile for physicians to provide the materials suggested for patients easily available, such as a napkins and flashlight, in order to demonstrate aspects of the medical exam on themselves if patients are experiencing difficulty [2]. Having access to a high-quality network signal or Wi-Fi is also vital [2]. Although a virtual examination may lack the needed elements of dynamic testing and diagnosis. Telemedicine necessitates that physicians use available resources to enhance the quality and outcome of patient’s remote treatment [27]. Setting up for an optimal consultation includes preparation by the patient. Thus, when booking the virtual appointment, patients may send a virtual visit checklist to the physicians and are encouraged to approve a number of items before the virtual examination [27]. Patients are advised to connect to telemedicine application to confirm their microphone and camera settings are working properly prior to the virtual consultation. Other checklist may include proper lighting and location, positioning of device camera, and clothing to allow for proper visibility and examination [27]. During the virtual consultation there is need for steady viewing. As such the camera of the patient/physician should be motionless and not held by hand during the virtual session. If patient is using a mobile device, the camera should be securely place in a position that can allow for clear view [27].

To ensure privacy both patient and physician should take the virtual consultation in a quiet space with reduced background noise so they can speak privately. It can be supportive to have someone who is trusted available to help with positioning of the camera [27]. The physician should keep it simple and provide a downloadable PDF guide for patients that can be delivered synchronously in real time or asynchronously in video link or another media format [4]. Furthermore, the physician can connected technology such as blood pressure monitors, weighing scales, and digital trackers to monitor patient [4]. Physician should clearly identify him/herself by full name, medical title, and medical affiliation. After which the physician should verify the patient’s complete names and date of birth before starting the virtual session. As the session starts the physician should speak clearly and pause recurrently to answer patient questions [17].

### Challenges and Recommendations for Use of Telemedicine

Telemedicine provide rapid access to medical-care remotely during health emergency [15]. Although, telemedicine can aid in remote assessment (triage) and continuity of medical-care, it is a disruptive process [28]. Several physicians had concerns regarding patient privacy policies, or whether telemedicine assessment was acceptable or meet the needed standards for a complete medical examination [25]. Thus, there is need to consider the challenges that may impact the successful use of telemedicine such as patient’s data confidentiality which must be established. Also, the effectiveness of telemedicine depends on the quality of the images and video. Thus, effective deployment of telemedicine requires availability of good infrastructure for both patient and physician. At times some diagnosis may be difficult to perform virtually [3]. Thus, it is also important that virtual software deployed for telemedicine should be user-friendly and also provide access to online assistance for patients with low technological proficiency [29].

Respectively, while telemedicine is gradually delivered through smart devices, the technology usually requires both the patient and physician to learn how to use a theses platform [12]. Also, medical practitioners may require knowledge and upskilling to be able to use virtual technology and equipment. Medical practitioners who already have prior knowledge in using virtual platforms could provide training [17], and support to other novel staff users [4]. Thus, there is need to provide training to physicians in using telemedicine [15, 30]. Accordingly, findings from Li and Jalali [29] revealed out of 5517 only 447 (8.1%) of physicians used provided telemedicine platform regardless of its benefits. This finding suggests the need to provide training to help physicians deliver medical-care remotely. Likewise, older patients are least likely to use telemedicine and virtual applications [29]. To address this issue there is need to educate patients so that they can be aware of virtual healthcare solutions and their associated benefits. Thus, telemedicine can be publicized through social media and other pervasive platforms to create awareness [29].

Most developing countries may not be able to fully adopt telemedical specifically in remote and rural areas due to low penetration of smart devices use and low expansion of 3G/4G internet networks [15]. Moreover, there is lack of fully designed legal framework to regulate the use of innovative IT solutions such as telemedicine in healthcare [3]. Also, in many developing countries there is lack of legislation that support telemedicine [21, 26]. In developing countries, the availability of adequate health facilities is an issue. Thus, governments should support and fund the health care systems in establishing telemedicine, laws and regulations needed. This will help to legislate and integrate telemedicine into the traditional health system [26]. Also, there is need to develop laws and upgrade technological infrastructures [23] and provide guidelines to address ethical and legal barriers to manage the use of telemedicine during health crises [26].

Additionally, in using telemedicine medical practitioners are required to adhere to local telemedicine practice regulations which impedes the provision of medical-care outside or across other states [31]. Also, financial barrier to patient using telemedicine may be a factor due to high treatment fee or inadequate insurance coverage or reimbursement for telemedicine will highly discourage potential users [29]. But, findings from Li and Jalali [29] suggest that financial barrier was not an issue and did not play a role since all COVID-19 related consultations are freely provided in China. Likewise, in the US, Medicaid, Medicare, and commercial healthcare plans have currently waived payment for telemedicine services in an attempt to help manage COVID-19. Thus, it is recommended that other countries help to eliminate or reduce financial burdens regarding use of telemedicine for treatment during health crises particularly for low income earners [29]. Therefore, current obsolete reimbursement and payment structure is an issue [3]. Accordingly, a mechanism needs to be provided to reimburse physicians for work done remotely [4].

Furthermore, as reported by [32], technical issue linked to internet access [24], Wi-Fi signal and bandwidth connectivity [25] impact the use of telemedicine. Thus, in some situation some nurses and physicians initially preferred using phone call for consultation as there was not stable enough signal for videoconference. An effective telemedicine requires reliable access to seamless data connection [17]. Likewise, there is need for virtual systems that provide real-time data that are translated for patients and medical practitioners without language barriers [17]. Furthermore, there have been recent security issues regarding the vulnerability of some virtual applications such as Zoom. In which users of the platform have been hijacking during video conferencing. Thus, raising concerns for privacy issues [33]. To address such issues a series of security assessments should be employed by informing patients to use the latest version of Zoom or update Zoom client. This will help to fix the vulnerabilities faced by enforcing end-to-end encryption. Likewise, physicians should use Zoom client with a corporate account to get enterprise security features. Additionally, password should be set for every treatment consultation meeting and the consultation details and password should be solely disclosed to physician and patient only. Lastly, the lock functionality should be enabled once physician and patient have joined the consultation session to avoid unauthorized intrusion [33].

## Discussion

Evidently, telemedicine and virtual platforms have the potential to help in addressing large-scale outbreaks and emergencies in highly uncertainty settings [8]. Accordingly, findings from [32] suggest that within the first 2 weeks of stay-at-home order, the number of telemedicine services increased to about 86% or higher in US, except for the hospital in Fayetteville, North Carolina, where telehealth consultations increased from 2% to 24%. Findings from Li and Jalali [29] suggest that telemedicine provide an effective triage, screening, and treatment method during the COVID-19 pandemic. Where, Li and Jalali [29] deployed an online platform to reduce the number of in-person visits thereby lessening face-to-face contact among patients and physicians, in reducing infection transmission. Findings from this study suggest that telemedicine and virtual care can be used by physicians to provide needed medical-care to patients during the pandemic and beyond.

Furthermore, findings from Triantafillou and Rajasekaran [28] suggest that telemedicine allows for examination of a patient’s health. It also helps to educate patients virtually on physical examination changes and symptom that should prompt a discussion with their physicians. Similarly, results from Patel et al. [34] indicated that patient stored heath information can provide guidance for future examination. However, it is to be noted that some hospitals and clinics in some countries may not have the resources to manage the surge in telemedicine and virtual patient care [21]. Health care policy makers can employ telemedicine due to the potential role it provides. Thus, deploying telemedicine could be a promising approach throughout this pandemic, given the number of out-patients in need of medical-care.

## Conclusion

Use of telemedicine to provide medical-care is not new. It has been previously utilized to connect with rural areas and deliver medical-care to patients remotely with satisfying results. Telemedicine technology is widely available, low-cost, and widely accepted by physicians and patients [23, 35]. With the emergence of COVID-19, cities being in lockdown from infection, the use of telemedicine by physicians and patients’ as treatment approach is necessary [18]. This current study discusses the current legislations for use of telemedicine and virtual applications during COVID-19 era, guidelines for use of telemedicine and virtual applications, and challenges and recommendations for use of telemedicine. Telemedicine is still an integral step in the right direction as medical practitioners are deploying innovate approaches to manage the COVID-19 situation [18].

Nevertheless, due to the evolving literature on this research area, it is likely that some studies published have not been included in the review. Secondly, the search terms used may not be exhaustive to get more studies similar to telemedicine. Future works will encompass retrieving more literature on how to improve telemedicine usage. Telemedicine provides a safe, accessible, and convenient medical-care. While, telemedicine is faced with many issues such as such availability of necessary infrastructure, inadequate funds, lack of experience, etc. Findings from this study provides insights to guide medical practitioners as they employ telemedicine to increase resilience to future health crises.
